# A Theoretical Study of the Binding of [Re_6_Se_8_(OH)_2_(H_2_O)_4_] Rhenium Clusters to DNA Purine Base Guanine

**DOI:** 10.3390/ma8073938

**Published:** 2015-06-29

**Authors:** Leonor Alvarado-Soto, Rodrigo Ramirez-Tagle

**Affiliations:** Laboratorio de Bionanotecnologia, Universidad Bernardo O’Higgins, General Gana 1780, Chile; E-Mail: rodrigoramireztagle@gmail.com

**Keywords:** rhenium clusters, anticancer, density functional theory, guanine binding

## Abstract

Hexanuclear rhenium complexes are promising candidates for use as antitumor drugs. However, to date, there has been no investigation into the nature of their binding to DNA. In this study, density functional theory (DFT) was used to examine the binding of [Re_6_Se_8_(OH)_2_(H_2_O)_4_] to the DNA purine base guanine. The geometrical structures of cluster-guanine adducts in water were modeled at the zero order regular approximation (ZORA)-PW91 level. Calculating the bond energies allowed us to compare the *cis* and *trans* forms of the cluster, and a possible manners of interaction between [Re_6_Se_8_(OH)_2_(H_2_O)_3_] clusters and DNA was obtained and explained.

## 1. Introduction

Recently, there has been a great deal of research into new materials based on inorganic clusters due to their luminescence and redox properties, as well as other attractive features [1,2,3,4,5,6,7]. An example of a useful inorganic cluster is a hexanuclear species with the general formula M_6_X_8_L_6_, where M = Re, Mo, or W; X = Cl, Br, I, S, Se, or Te; L = F, Cl, Br, I, CN, NCS, OH, H_2_O, dendrons, pyridines, *etc*. [8,9].

Current studies indicate that hexanuclear rhenium clusters have antitumor properties, which suggests that these compounds have potential as novel heavy-metal drugs [10,11,12,13,14,15]. For example, compounds with the general formula [Re_6_Se_8_I_6_]^3−^, can induce 100% cell death in cancerous skin and liver cell lines while leaving healthy cells unaffected [12]. Additionally, rhenium-based clusters of the type [Re_6_Se_8_(H_2_O)*_n_*(OH)_6−*n*_]*^n^*^−4^ have been shown to antiproliferative activity in cervical cancer cell lines (IC_50_ = 262.3 µM) [10].

Most heavy-metal-based drugs interact with DNA and, thus, alter the metabolism of tumor cells in such a way that they cause cell death. In general, these interactions involve hydrolysis of the compound and direct formation of covalent bonds with nucleotide bases—Principally between the metal and the N7 atom of adenine or guanine [16,17,18,19]. However, the antitumor activity of rhenium clusters appears to depend on their axial substituents, which implies that their interaction with DNA could be through a different, as yet unknown, mechanism [10,11,12,14].

**Figure 1:**
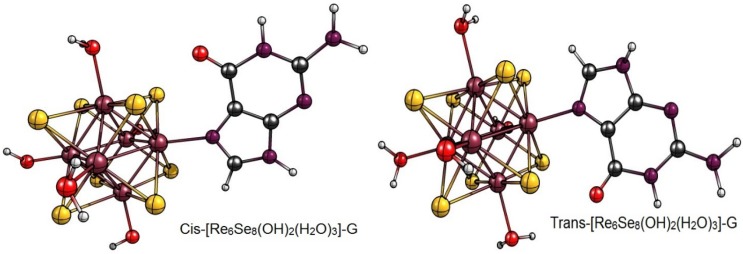
Molecular structure of *cis*-[Re_6_Se_8_(OH)_2_(H_2_O)_3_]-guanine and *trans*-[Re_6_Se_8_(OH)_2_(H_2_O)_3_]-guanine complexes.

In addition, the strength of the interaction between a drug and DNA is directly related to antitumor capacity; therefore, careful characterization of potent compounds provides vital information for the design and synthesis of new compounds [20,21]. One means of characterizing the interactions between inorganic clusters and target biomolecules is through the use of theoretical methods based on density functional theory (DFT) [22]. In this study we have used DFT to examine the binding modes of the hexanuclear rhenium cluster-guanine complexes *cis*- and *trans*-[Re_6_Se_8_(OH)_2_(H_2_O)_3_]-G (Figure 1) with the goal of elucidating their mechanism and informing the design of more efficacious compounds in the future. For comparison, we also examine a common *cis*-Pt drug.

## 2. Results and Discussion

The hexanuclear cluster-G complexes described in this study would be very stable under physiological conditions; therefore it would be difficult to examine this type of covalent interaction with DNA, and there would be no interaction via intercalation [12]. Clusters of the type [Re_6_Se_8_(H_2_O)*_n_*(OH)_6−*n*_]*^n^*^−4^ are likely to establish a covalent interaction with nitrogenous bases; moreover, at physiological pH, they have hydroxyl groups and water molecules as axial substituents, as shown in previous theoretical and experimental studies [23,24].

The results of the full geometric optimization computations by the DFT method at ZORA-PW91-COSMO [25,26,27,28] level are appropriate for this system, and on the basis of the computed geometries of the complexes, we can conduct a structural analysis of the binding between the hydrated rhenium species to DNA model bases at higher basis set level [22,29,30]. Since there are no yet reported measurements of the Guanine cluster interactions, our results reported here may have a predictive character. The binding energies for rhenium cluster complexes of guanine are summarized in Table 1. The observed distances between N7 and rhenium are about 2.18 Å, which is similar to the Re-N distances observed in cyclic systems (about 2.15 Å) (Figure 1) [3,14].

**Table 1:** Calculated binding energies for the examined rhenium cluster-guanine complexes.

Complexes	Binding Energy
*Cis*-[Re_6_Se_8_(OH)_2_(H_2_O)_3_]-G	−869.25 kcal/mol
*Trans*-[Re_6_Se_8_(OH)_2_(H_2_O)_3_]-G	−332.03 kcal/mol
*Cis*-[Pt(NH_3_)_2_(H_2_O)]-G^2+^	−315.17 kcal/mol

As shown in Table 1, the *cis* conformation of [Re_6_Se_8_(OH)_2_(H_2_O)_3_]-G was found to be 537.22 kcal/mol more energetically favorable than *trans*-[Re_6_Se_8_(OH)_2_(H_2_O)_3_]-G. By way of comparison, the binding of *cis*-Pt[(NH_3_)_2_(H_2_O)]-G^2+^ was 16.86 kcal/mol less favorable than *trans*-[Re_6_Se_8_(OH)_2_(H_2_O)_3_]-G.

As shown in Table 2, the HOMO, HOMO-1, LUMO, and LUMO+1 for the clusters and the cluster complexes are localized at the cluster core [Re_6_Se_8_]^2+^. The nature of the molecular orbitals changes significantly upon complex formation and charge redistribution takes place. Mulliken charge analysis of the isomeric configurations of the [Re_6_Se_8_(OH)_2_(H_2_O)_3_]-G cluster complexes reveals a small charge transfer during complex formation (Table 3).

**Table 2:** Molecular orbital diagrams of the examined rhenium cluster-guanine complexes.

*Cis*-[Re_6_Se_8_(OH)_2_(H_2_O)_3_]	*Trans*-[Re_6_Se_8_(OH)_2_(H_2_O)_3_]	*Cis*-[Re_6_Se_8_(OH)_2_(H_2_O)_3_]-G	*Trans*-[Re_6_Se_8_(OH)_2_(H_2_O)_3_]-G	Guanine
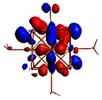 HOMO -1	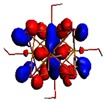 HOMO -1	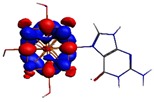 HOMO -1	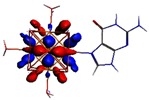 HOMO-1	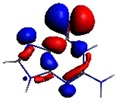 HOMO-1
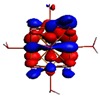 HOMO	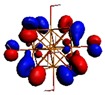 HOMO	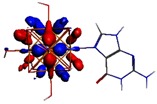 HOMO	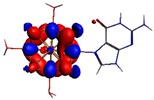 HOMO	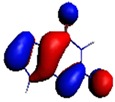 HOMO
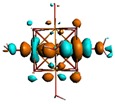 LUMO	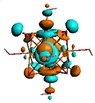 LUMO	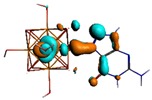 LUMO	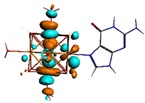 LUMO	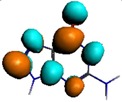 LUMO
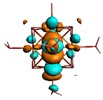 LUMO +1	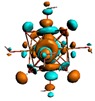 LUMO +1	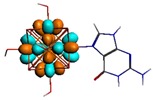 LUMO +1	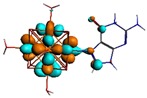 LUMO +1	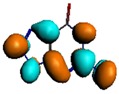 LUMO +1

**Table 3:** The Mulliken charges of cluster, guanine and cluster-guanine complex.

*Cis*-[Re_6_Se_8_(OH)_2_(H_2_O)_3_]	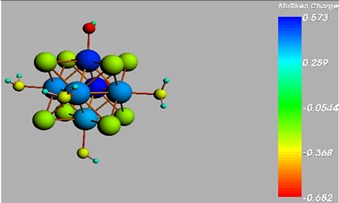
*Trans*-[Re_6_Se_8_(OH)_2_(H_2_O)_3_]	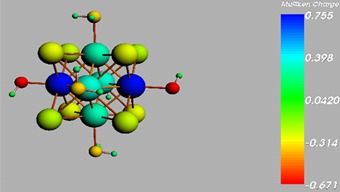
*Cis*-[Re_6_Se_8_(OH)_2_(H_2_O)_3_]-G	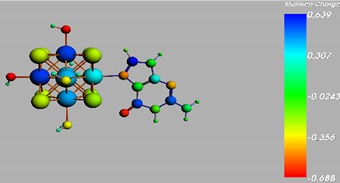
*Trans*-[Re_6_Se_8_(OH)_2_(H_2_O)_3_]-G	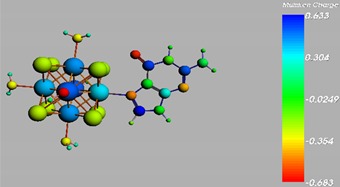
Guanine	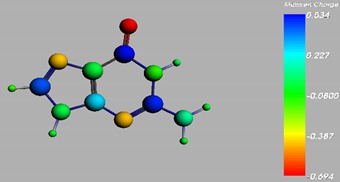

## 3. Experimental Section 

Our calculations were carried out using Amsterdam Density Functional (ADF) code [28]. The scalar and spin-orbit relativistic effects were incorporated using the zero order regular approximation (ZORA) [25]. All molecular structures were fully optimized using the analytical energy gradient method implemented by Verluis and Ziegler employing the local density approximation (LDA) within the Vosko-Wilk-Nusair parameterization for local exchange correlations. Solvation effects were modeled with a conductor-like screening model for real solvents (COSMO) using water as the solvent [27]. Cluster geometry was optimized and excitations energies were calculated using standard Slater-type-orbital (STO) basis sets with triple-zeta quality double plus polarization functions (TZ2P) for the all the atoms.

The analysis of bonding energetics was performed by a fragment approach [Cluster-Guanine] to the molecular structure of a chemical system. 

## 4. Conclusions

In this work, the binding of an antitumor rhenium cluster to G purine bases was studied using the DFT method. The theoretical results show that for the complex studied in this work, the *cis* adduct binds more effectively than the *trans* adduct. This suggests that the guanine N7 is the possible site of interaction. This work represents the first time that interactions between [Re_6_Se_8_(OH)_2_(H_2_O)_3_] clusters and DNA have been modelling one of the possible manners of interaction between [Re_6_Se_8_(OH)_2_(H_2_O)_3_] clusters and DNA, and the data provide some of the understanding that is needed to further improve this promising class of drug. 
